# Low Income, Younger Age, Female Sex, and Poor Mental Health Are Risk Factors for Diminished Access to Care Among Patients With Meniscus Tears

**DOI:** 10.1016/j.asmr.2024.101064

**Published:** 2024-12-12

**Authors:** Drew W. Barron, Vikram S. Gill, Sayi P. Boddu, Nathan C. Beckett, Sailesh V. Tummala, Anikar Chhabra

**Affiliations:** a Mayo Clinic Alix School of Medicine, Scottsdale, Arizona, U.S.A.; b Department of Orthopedic Surgery, Mayo Clinic, Phoenix, Arizona, U.S.A.

## Abstract

**Purpose:**

To use the All of Us database to investigate barriers to health care access for patients with meniscus tears and to examine how specific demographic, socioeconomic, and health-related factors independently influence their health care utilization.

**Methods:**

The National Institutes of Health All of Us database was queried for patient data between March 2017 and March 2023 to form a cohort of patients with meniscal tears. Patients with meniscal tears were identified using Systematized Nomenclature of Medicine clinical terms. All patients with self-reported meniscus tears were included, with no exclusion criteria to ensure a diverse representation of underrepresented individuals. Patient responses to the “Healthcare Access and Utilization Survey” were analyzed across 4 domains: “delayed care,” “could not afford care,” “skipped medication,” and “have not seen a provider in over one year.”

**Results:**

In total, 5,671 survey respondents with meniscal tears were included for analysis from a population of 436,000 eligible records. Among them, 27.7% reported delayed care, 23.9% were unable to afford care, 11.8% skipped medication, and 2.0% had not seen a provider in over a year. Patients with annual incomes under $50,000 were more likely to delay care (odds ratio [OR], 1.41; *P* < .001), be unable to afford care (OR, 1.73; *P* < .001), or skip medications (OR, 1.79; *P* < .001). Younger age than the cohort mean of 65.4 years, female sex, and poor physical and mental health were associated with impaired access in at least 1 care domain.

**Conclusions:**

Over one-fourth of patients with a diagnosis of meniscus tear who responded to the All of Us “Healthcare Access and Utilization Survey” reported difficulties with access to care across 4 domains, with those of low income, younger age, female sex, and poor mental or physical health experiencing significant barriers to care.

**Clinical Relevance:**

This research can inform strategies for equitable treatment of meniscal injuries by highlighting disparities in orthopaedic care access and utilization.

The meniscus plays a crucial role in knee function, including shock absorption, joint stability, load support, position sense, lubrication, and nutritional support.^1^^,^^2^ Meniscal tears are common across all ages, with an incidence between 0.6 and 0.7 per 1,000 person-years in the general populace, and impose a sizable annual cost burden of nearly $5 billion on the US health care system.^3^ Meniscal tears can be managed surgically or nonsurgically, with determination based on tear location, type, and size as well as the patient’s age, overall knee condition, time since injury, activity level, and care goals.^4^ Regardless of the management approach, individuals with meniscal tears require timely access to care to monitor injury progression and determine the best approach to management.

In a survey of American Orthopaedic Association members, 68% of respondents indicated that at least some evidence of disparity exists in orthopaedic care.^5^ White patients, for example, were found to have a higher rate of undergoing meniscal surgery (58.6%) than African American (35.4%), Hispanic (34.9%), or Asian (39.4%) patients.^6^^,^^7^ Furthermore, patients without insurance or those with government-subsidized insurance were less likely to have surgery than those with private insurance or worker’s compensation claims.^6^^,^^7^ Females were also found to have a lower rate of surgical intervention for the management of meniscal tears.^7^ Advancement in understanding the disparities affecting meniscal surgery and the underlying factors that influence care access may help guide strategies to improve care equity, shape future health policy, and ensure patients receive appropriate treatment strategies for their condition regardless of background. Unlike prior studies that focus on surgical rates alone, our study aims to broaden this understanding by examining how demographic, socioeconomic, and health-related factors impact overall access to surgical and nonsurgical care options for meniscal injuries. By identifying access barriers more comprehensively, we seek to inform strategies that address inequities in the full spectrum of care for patients with meniscal tears.

Prior studies have reported risk factors associated with lower rates of meniscectomy among well-represented populations, and these findings focus primarily on surgical intervention decisions rather than overall access to orthopaedic care.^3^^,^^4^ A lower rate of meniscectomy may reflect clinical decision-making based on patient or injury characteristics,^2^^,^^3^ whereas diminished access to care encompasses broader barriers. This gap in the literature points to a need for research that goes beyond surgical outcomes to examine how socioeconomic, demographic, and health-related factors independently affect access to initial and ongoing care for meniscal injuries, providing a foundation for more equitable health care practices in orthopaedics. This study utilizes respondent data from the All of Us database to explore factors affecting health care access for patients with meniscus tears. The National Institutes of Health (NIH) launched the All of Us database in 2018 to serve as a deidentified data set for secondary research studies on a standardized diverse population focused on human health and disparities.^8^^,^^9^ The program is designed to recruit participants who voluntarily sign up, focusing on including groups historically underrepresented in biomedical research. Recruitment efforts promote data diversity through partnerships with community organizations, targeted outreach, and engagement initiatives aimed at racial and ethnic minorities, lower-income individuals, rural populations, and other groups with limited access to health care.^8^ Once enrolled, participants provide informed consent and contribute health information through multiple channels, including electronic health records (EHRs), surveys on health and lifestyle factors, and physical measurements.^8^

To support research on health disparities, the All of Us program collects data from a demographically diverse participant pool, intentionally recruiting individuals from populations that are often underrepresented in biomedical studies.^8^ This diverse data set, updated as participants contribute new information or as their EHRs capture health care interactions, provides an opportunity to study disparities in health care.^8^ By utilizing this resource, this study promotes equity in the treatment of meniscal tears and aligns with the overarching goals of the US Department of Health and Human Services Healthy People 2020, which seeks to foster health equity, eradicate disparities, and enhance the well-being of all communities.^10^

The purposes of this study were to use the All of Us database to investigate barriers to health care access for patients with meniscus tears and to examine how specific demographic, socioeconomic, and health-related factors independently influence their health care utilization.

## Methods

### Data Source

Patient data for the analysis of patients with meniscus tears were sourced from the All of Us database from the NIH for retrospective analysis. The source database comprises more than 1,201,000 registrants to the online portal, 545,500 participants who have completed the program’s initial steps, and 436,000 electronic health records between May 2017 and May 2023.^11^ All of Us data include 45% racial and ethnic minorities as well as 80% of the participants being previously underrepresented in biomedical research.^11^ Given the retrospective nature of the study and deidentified patient data set, approval from our local institutional review board was deemed exempt for this study.

### Patient Cohort

To build a historical cohort for study, the All of Us database was parsed for all registered patients diagnosed with meniscus tear who completed the “Healthcare Access and Utilization Survey” between May 2017 and May 2023. The survey is based on the National Health Interview Survey, a validated patient-reported outcome measure that gathers information on patient demographics, socioeconomic status, and health-related data, including medication utilization and care access.^12^ To identify patients with meniscus tears for review, patients were recognized using the Systemized Nomenclature of Medicine clinical terms for meniscus tears. All patients who self-reported their diagnosis of meniscus tear were included rather than clinically confirmed, which may affect the accuracy of the diagnosis. Given the retrospective and deidentified nature of this study’s data set, it was not possible to control for patients with potentially confounding conditions, such as anterior cruciate ligament tears or osteoarthritis. No explicit exclusion criteria were applied to respect the diversity of the population and capture a wide representation of individuals with this condition. Physical and mental health summary scores were derived from the Patient-Reported Outcome Measurement Information System (PROMIS) project short-form survey, and normalized responses were utilized to build a representative and valid cohort.^13^

### Outcome Measures

The primary outcome measured in this study was self-reported patient data from patients with meniscus tears in the “Healthcare Access and Utilization Survey” in the All of Us database on their ability to access and afford health care. For the analysis of meniscus tears, the survey includes patient responses across 4 care domains: delayed medical care, could not afford care, skipped medications, and over 1 year since seeing a health care provider. While these domains are not exclusive to meniscal tear treatment, they were included to capture broader patterns of health care access and utilization that could influence overall health outcomes for individuals with meniscus tears. For the purposes of this study, we refer to 182 “older age” as ages above the cohort mean of 65.4 years and “younger age” as ages below this 183 mean.

Responses to the 3 domains—delayed care, skipped medications, and inability to afford care—were represented through responses to more than 1 survey question. Given the presence of multiple related survey questions to each care domain, any “yes” answer was considered a valid measure of the patient experiencing a barrier to that care domain for this retrospective analysis. Survey respondents were asked about care delays in 10 ways, such as “couldn’t get time off work,” “couldn’t get child care,” “couldn’t afford the copay,” and “you had to pay out of pocket for some or all of the procedure.”^14^ If a patient responded “yes” to any of the survey questions related to delays in obtaining medical care, including delays in seeing any doctor or health care provider rather than solely an orthopaedic specialist, the patient was coded as having experienced delayed medical care. These questions were grouped into a single outcome because each question clarified why the respondent delayed their medical care, but all questions indicated a delay in receiving medical care.^14^ This methodology was extended to skipped medications and inability to afford care, which also contained multiple related and representative questions. The survey questions that were considered for analysis in this study can be found published online on the All of Us website (https://www.researchallofus.org/data-tools/survey-explorer/healthcare-access-utilization-survey/).

After synthesizing survey respondent data, the 4 domains of care were compared in the meniscus tear cohort to evaluate the impact of the presence of a meniscus tear on access to medical care. For further analysis, the study compares factors between patients who reported poor access to care, defined as answering “yes” to any of the 4 domains, and those who reported adequate access to care.

### Statistical Analysis

For each access-to-care domain of delayed medical care, inability to afford care, skipped medications, and over 1 year since seeing a health care provider, a univariate logistic regression analysis was conducted to determine specific patient factors correlated with reduced access to care. For the purposes of this data analysis, statistical significance was defined as a *P* value of less than .05. After initial analysis, univariate outcomes that were deemed to be statistically significant (*P* < .05) were then utilized in further multivariable logistic regression. Data analysis was conducted on the All of Us website, utilizing the online workbench using R in Jupyter Notebooks. Jupyter Notebooks is an open-source data science and scientific computing tool developed by Project Jupyter and supported by the nonprofit organization NumFOCUS (https://jupyter.org).

## Results

### Patient Demographics

In total, 5,671 patients with meniscus tears were included in this retrospective analysis from a population of 436,000 eligible EHRs. The mean (SD) age of these patients was 65.4 (12.5), with a range of 20.8 to 90.4 years. Of the patients, 63.7% of patients were female (n = 3,614). Most respondents were White identifying (86.0%, n = 4,878). Only 36.1% of survey respondents reported income above $100,000 per year, and 26.1% of participants self-reported income less than $50,000 annually. Additionally, 36.8% of included patients with meniscus tears had an educational obtainment of less than a college degree (Table 1).

**Table 1:** Patient Demographics of the Meniscal Tear Cohort

Demographics	Meniscal Tear (n = 5,671)
**Age**	
Mean (SD)	65.4 (12.5)
Range	20.8-90.4
**Sex**	
Female	3,614 (63.7)
Male	2,057 (36.3)
**Race**	
Asian	77 (1.4)
Black or African American	532 (9.4)
Multiracial	84 (1.5)
White	4,878 (86.0)
**Ethnicity**	
Hispanic or Latino	77 (1.4)
Not Hispanic or Latino	5,533 (97.6)
**Income**	
>$200,000	677 (11.9)
$100,000-$200,000	1,375 (24.2)
$50,000-$100,000	1,525 (26.9)
$0-$50,000	1,481 (26.1)
**Education**	
College graduate or advanced degree	3,530 (62.2)
College but did not finish	1,459 (25.7)
High school graduate or General Education Development	558 (9.8)
Less than a high school degree	73 (1.3)

NOTE. Values are presented as n (%) unless otherwise indicated.

### Meniscus Tears and Care Access

Among the 5,671-patient cohort with meniscus tears, 27.7% (n = 1,570) of survey respondents reported delayed care, 23.9% (n = 1,355) reported inability to afford care, and 11.8% (n = 667) reported skipped medication. Additionally, 116 respondents (2.0%) self-reported that they had not seen or talked to a doctor or health care provider about their health in over 1 year.

### Demographics Associated with Poor Care Access

An income of less than $50,000 annually was associated with increased rates of delayed care in patients with meniscus tears (odds ratio [OR], 1.41; *P* < .001). In contrast, male sex (OR, 0.69; *P* < .001), older age (OR, 0.96; *P* < .001), and income in excess of $100,000 annually (OR, = 0.81, *P* = .010) were identified to be protective factors, with decreased rates of care delay in survey respondents with meniscal tears. Better physical (OR, 0.75; *P* < .001) and mental health (OR, 0.83; *P* < .001), as determined by the PROMIS surveys, also corresponded with decreased delays in care (Table 2).

**Table 2:** Multivariable Analysis of Meniscus Surgery and Demographic Factors as Predictors of Delayed Medical Care in Patients With Meniscal Tear

Variable	Odds Ratio	95% CI Lower	95% CI Upper	*P* Value
**Delayed care**				
Meniscus surgery	0.94	0.58	1.50	.78
Age	0.96	0.95	0.96	<.001∗
Male sex	0.69	0.60	0.80	<.001∗
White race	0.83	0.62	1.10	.20
Black race	0.79	0.56	1.11	.17
Income: <$50,000	1.41	1.20	1.66	<.001∗
Income: >$100,000	0.81	0.69	0.95	.010∗
Education: college	0.75	0.40	1.41	.37
Education: high school	0.62	0.32	1.19	.15
Education: < high school	0.81	0.36	1.81	.60
No partner	1.10	0.95	1.27	.19
Sexuality: not heterosexual	1.17	0.93	1.48	.17
PROMIS Physical	0.75	0.69	0.81	<.001∗
PROMIS Mental	0.83	0.77	0.89	<.001∗

NOTE. Only variables significant on univariate analysis were included in multivariable analysis for each domain.

PROMIS, Patient-Reported Outcomes Measurement Information System.

∗ *P* < .05.

The inability to afford care was associated with an income of less than $50,000 annually (OR, 1.73; *P* < .001). Protective factors against inability to afford care were the same as delays in care, with male sex (OR, 0.67; *P* < .001), age (OR, 0.97; *P* < .001), income over $100,000 (OR, 0.50; *P* < .001), better physical health (OR, 0.72; *P* < .001), and better mental health (OR, 0.79; *P* < .001) associated with an ability to medical care (Table 3).

**Table 3:** Multivariable Analysis of Meniscus Surgery and Demographic Factors as Predictors of Inability to Afford Care in Patients With Meniscal Tear

Variable	Odds Ratio	95% CI Lower	95% CI Upper	*P* Value
**Could not afford care**				
Meniscus surgery	0.60	0.35	1.04	.07
Age	0.97	0.97	0.98	<.001∗
Male sex	0.67	0.58	0.78	<.001∗
White race	0.79	0.55	1.14	.21
Black race	0.95	0.63	1.43	.80
Multiracial	1.09	0.60	2.00	.78
Income: <$50,000	1.73	1.37	2.17	<.001∗
Income: $50,000 to $100,000	0.88	0.70	1.11	.29
Income: >$100,000	0.50	0.39	0.64	<.001∗
Education: college	1.07	0.55	2.10	.84
Education: high school	0.94	0.47	1.88	.85
Education: < high school	0.80	0.35	1.85	.60
No partner	1.16	1.00	1.34	.06
Sexuality: not heterosexual	1.17	0.92	1.49	.19
PROMIS Physical	0.72	0.66	0.78	<.001∗
PROMIS Mental	0.79	0.73	0.86	<.001∗

NOTE. Only variables significant on univariate analysis were included in multivariable analysis for each domain.

PROMIS, Patient-Reported Outcomes Measurement Information System.

∗ *P* < .05.

Income less than $50,000 (OR, 1.79; *P* < .001) resulted in a higher odds of self-reported skipped medications. Male sex (OR, 0.57; *P* < .001), age (OR, 0.98; *P* < .001), income over $100,000 (OR, 0.55; *P* < .001), better physical health (OR, 0.66; *P* < .001), and better mental health (OR, 0.89; *P* = .023) were associated with better adherence to medication regimen (Table 4).

**Table 4:** Multivariable Analysis of Meniscus Surgery and Demographic Factors as Predictors of Skipped Medications or Over 1 Year Since Seeing a Provider in Patients With Meniscal Tear

Variable	Odds Ratio	95% CI Lower	95% CI Upper	*P* Value
**Skipped medications**				
Meniscus surgery	0.68	0.33	1.37	.28
Age	0.98	0.97	0.98	<.001∗
Male sex	0.57	0.46	0.70	<.001∗
White race	1.01	0.62	1.64	.97
Black race	0.99	0.58	1.68	.97
Multiracial	1.17	0.55	2.47	.69
Income: <$50,000	1.79	1.46	2.19	<.001∗
Income: >$100,000	0.55	0.43	0.70	<.001∗
Education: college	1.59	0.92	2.74	.10
Education: high school	1.23	0.68	2.20	.49
No partner	1.00	0.82	1.20	.97
Sexuality: not heterosexual	1.07	0.80	1.44	.65
PROMIS Physical	0.66	0.60	0.74	<.001∗
PROMIS Mental	0.89	0.81	0.98	.023∗
**>1 year since seeing provider**				
Meniscus surgery	1.76	0.72	4.30	.21
Age	0.96	0.95	0.97	<.001∗
Male sex	1.79	1.22	2.61	.003∗
Income: >$100,000	1.33	0.91	1.96	.15
PROMIS Physical	1.46	1.19	1.79	<.001∗

NOTE. Only variables significant on univariate analysis were included in multivariable analysis for each domain.

PROMIS, Patient-Reported Outcomes Measurement Information System.

∗ *P* < .05.

In patients with meniscus tears, male sex (OR, 1.79; *P* = .003) and better PROMIS physical health scores (OR, 1.46; *P* < .001) were associated with a higher likelihood of the patient having gone more than a year without seeing a provider. Increased age was a protective factor, with older age identified to have a higher likelihood of receiving medical care in the past year (*P* < .001) (Table 4).

## Discussion

The most important finding of this study among our cohort of 5,671 patients with meniscus tears who completed the “Healthcare Access and Utilization Survey” in the All of Us database was that over a quarter of respondents encountered significant barriers to health care access. In total, 27.7% reported delays in care, 23.9% were unable to afford treatment, 11.8% skipped medications, and 2.0% had not seen a health care provider in over a year. Patients who were female sex, younger age, or low income or had poor mental or physical health were associated with a higher likelihood of an inability to access or afford adequate medical care. The large, information-rich open-enrollment NIH All of Us dataset allowed the study to identify associations between patient demographics, socioeconomic status, and health-related factors that influence the access to care of patients with meniscal tears and identify barriers to accessing and utilizing health care services among them. The associations identified in this study underscore the potential need for targeted interventions to address health care disparities and improve access for vulnerable patient populations with meniscus tears; however, these findings are specific to meniscal injuries and may not necessarily extend to other orthopaedic conditions.

Among our meniscal tear cohort, 27.7% of patients reported delaying care for reasons related to lack of transportation, nervousness regarding seeing a health care provider, inability to get time off of work, inability to find child care, provision of care to an adult, inability to afford the care, or out-of-pocket cost burden of care. A delay in receiving adequate care for meniscal tears can compromise immediate well-being and may worsen long-term outcomes by reducing the likelihood of a successful repair or delaying the restoration of meniscal function; for tears treated nonsurgically or with partial meniscectomy, delays primarily impact the patient’s quality of life until treatment. If left without requisite treatment, meniscal tears can negatively modify joint dynamics and increase the rate of knee cartilage degeneration. Patients who lack insurance or have public insurance have increased nonvoluntary injury to referral time, injury to evaluation time, and injury to surgical repair time.^15^ Untreated meniscal tear may lead to osteoarthritis or a total knee replacement, a more costly and resource-intensive procedure.^16^

Patients who delay care due to inadequate access may contribute to the growing annual cost burden of $5 billion for meniscus injuries every year in the United States.^3^ To illustrate this point, patients who undergo prompt partial meniscectomy have 24-month direct costs of $4,488, while nonoperative patients with nonobstructing meniscus tears may face considerably higher costs if nonoperative management ultimately fails and they require more intensive interventions.^3^ Given that 23.9% of study participants in the meniscus tear cohort could not afford their current health care expenditures, addressing care access and providing timely surgical intervention is crucial. An unstable meniscus, if not properly treated, may lead to degenerative arthritis progression due to unstable flaps causing mechanical abrasion or meniscal root tears destabilizing the meniscal body, thereby limiting its ability to function in hoop stress absorption. This could ultimately result in increased long-term costs due to joint breakdown and the need for more extensive and costly treatments in an already disadvantaged population.

Further analysis is necessary to determine the impact of a patient’s ability to pay for their health care costs and the surgical approach of choice, with consideration of meniscal repair and meniscectomy. Meniscal repair is associated with higher day-of-surgery, 9-month, and 2-year costs compared to meniscectomy; however, repair is considerably more cost-effective due to reduced rates of future total knee arthroplasty and osteoarthritis.^17^^,^^18^ With consideration for patients with low income or reduced access to care, it is, however, imperative to note that meniscal repair does have a higher failure rate (19.1%) than partial meniscectomy, which may complicate outcomes for patients who delay care or do not present for proper follow-up evaluation.^19^ Another critical factor to consider is the recovery time postsurgery; meniscectomy typically involves a 4- to 6-week recovery period with minimal immediate postoperative limitations, whereas meniscal repair requires a significantly longer recovery period of 3 to 6 months, often with strict weightbearing restrictions immediately after surgery.^20^^,^^21^ Therefore, it remains important to consider demographic risk factors, such as socioeconomic status and access to care, alongside clinical factors when determining the optimal treatment strategy, as these variables may influence patient outcomes and follow-up adherence.

Previous studies have identified several risk factors associated with a lower rate of meniscectomy, which includes factors such as female sex, lower socioeconomic status, and racial/ethnic minority status.^6^^,^^7^ These studies suggest that such populations may experience lower rates of meniscectomy due to barriers in accessing specialty orthopaedic care, lower likelihood of insurance coverage, and physician biases regarding surgical intervention for certain demographic groups. However, unlike our study, prior research has primarily focused on surgical decision-making rather than overall access to orthopaedic care, underscoring a gap in understanding how health care access impacts nonsurgical management and outcomes in meniscal injuries. The findings of this study align with prior studies in identifying female sex and lower socioeconomic status as factors linked to reduced health care access for patients with meniscal tears, although we did not observe race as a significant factor in this study. By focusing on overall access to care rather than only surgical intervention, our study adds to the literature by demonstrating that these barriers impact both surgical and nonsurgical management, potentially contributing to disparities in long-term outcomes for patients with limited access.

This study identified female sex, younger age, low income, and poor physical or mental health to be independent risk factors predisposing patients in the meniscal tear cohort to self-reported inadequate access to care. In a review of 387,333 meniscectomies and 23,640 meniscus repairs between 2005 and 2011, females were found to comprise 39% of the meniscal repairs and 43% of the meniscectomies.^22^ Females represent a large share of the population undergoing meniscal surgery and are associated with a significantly increased risk of delayed care and inability to afford care. It is necessary to consider sex as an essential confounding variable that may impact a patient’s continuum of orthopaedic care. In this study, “younger age” was associated with reduced health care access, potentially due to lower insurance coverage rates and affordability challenges that younger individuals commonly face, which can hinder their ability to afford necessary care.^23^^,^^24^ Additionally, younger patients may deprioritize medical attention due to work or personal responsibilities, further contributing to delays in seeking timely care.^25^

Patients with a diagnosis of meniscus tear who were low income, younger age, or female sex or had poor mental or physical health were found to be more susceptible to decreased access to adequate care. This retrospective cohort analysis highlights specific factors influencing access to care for these patients, warranting further research and targeted interventions to reduce disparities and promote equitable treatment. Addressing these factors is essential for improving health care access and outcomes in vulnerable populations with meniscus tears. Future efforts should focus on mitigating these disparities to ensure that treatment opportunities and health care resources are accessible to those most in need. Although this study focuses on patients with meniscus tears, the findings have broader implications, suggesting a need for orthopaedic research that identifies and addresses barriers to adequate care across the full spectrum of orthopaedic conditions.

### Limitations

The use of a retrospective database has inherent limitations, including the precision of data and the ability to elucidate the temporal relationship between data. While the use of logistic univariate and multivariate regression can establish statistically significant associations between patient-specific factors, it cannot establish causality, nor can it provide contextualization between interplaying demographic, socioeconomic, and health factors that drive care access. Additionally, while self-reported data greatly reflect the patient experience and their interpretation of care access, the possibility of selection bias or question misinterpretation may skew outcomes. Furthermore, it is essential to consider the recruitment approach of the All of Us database, which may only partially represent the broader US population due to its open, nonprobabilistic sampling method. This recruitment strategy could introduce certain biases, particularly if individuals with specific demographic or health characteristics are more inclined to participate. Additionally, the absence of detailed information on the type of meniscal tear (e.g., stable horizontal tears vs unstable flap, radial, or root tears) limits our ability to differentiate the clinical symptoms and access challenges specific to each tear type, as horizontal tears are often asymptomatic. Given the standardized and deidentified nature of the data, it was impossible to account for factors such as the insurance status or geographic location of survey respondents. As an observational study, there is a possibility of unmeasured confounding variables influencing the results, which cannot be fully accounted for in this analysis. While the diversity of the All of Us cohort—with 80% of participants from groups traditionally underrepresented in medical research—is a strength in addressing health disparities, this focus may limit the generalizability of the data set and study findings to other patient populations.

## Conclusions

Over one-fourth of patients with a diagnosis of meniscus tear who responded to the All of Us “Healthcare Access and Utilization Survey” reported difficulties with access to care across 4 domains, with those of low income, younger age, female sex, and poor mental or physical health experiencing significant barriers to care.

## Disclosures

The authors declare the following financial interests/personal relationships which may be considered as potential competing interests: A.C. receives speaking and lecture fees from Arthrex and is a consultant or advisor for Zimmer Biomet. All other authors (D.W.B., V.S.G., S.P.B., N.C.B., S.V.T.) declare that they have no known competing financial interests or personal relationships that could have appeared to influence the work reported in this paper.

## References

[1] Bland-Sutton J. (1897). Ligaments: Their nature and morphology. Bristol Med Chir J.

[2] Arner J.W., Ruzbarsky J.J., Vidal A.F., Frank R.M. (2022). Meniscus repair part 1: Biology, function, tear morphology, and special considerations. J Am Acad Orthop Surg.

[3] Adams B.G., Houston M.N., Cameron K.L. (2021). The epidemiology of meniscus injury. Sports Med Arthrosc Rev.

[4] Espejo-Reina A., Aguilera J., Espejo-Reina M.J., Espejo-Reina M.P., Espejo-Baena A. (2019). One-third of meniscal tears are repairable: An epidemiological study evaluating meniscal tear patterns in stable and unstable knees. Arthroscopy.

[5] Adelani M.A., O’Connor M.I. (2017). Perspectives of orthopedic surgeons on racial/ethnic disparities in care. J Racial Ethn Health Disparities.

[6] Schairer W.W., Nwachukwu B.U., Lyman S., Allen A.A. (2018). Race and insurance status are associated with surgical management of isolated meniscus tears. Arthroscopy.

[7] O’Donnell R., Lemme N., Brodeur P., Gil J., Cruz A. (2021). Racial, socioeconomic, and demographic disparities in the management of meniscal tears. Orthop J Sports Med.

[8] National Institutes of Health All of Us Research Hub. National Institutes of Health. https://www.researchallofus.org/.

[9] Mayer C.S., Huser V. (2023). Learning important common data elements from shared study data: The All of Us program analysis. PLoS One.

[10] US Department of Health and Human Services Healthy People 2020. https://health.gov/our-work/national-health-initiatives/healthy-people/healthy-people-2020.

[11] National Institutes of Health All of Us data snapshots. https://www.researchallofus.org/data-tools/data-snapshots/.

[12] Blackwell D.L., Lucas J.W., Clarke T.C. (2014). Summary health statistics for U.S. adults: national health interview survey, 2012. Vital Health Stat 10.

[13] Hays R.D., Bjorner J.B., Revicki D.A., Spritzer K.L., Cella D. (2009). Development of physical and mental health summary scores from the Patient-Reported Outcomes Measurement Information System (PROMIS) global items. Qual Life Res.

[14] National Institutes of Health All of Us Healthcare Access and Utilization Survey. https://www.researchallofus.org/data-tools/survey-explorer/healthcare-access-utilization-survey/.

[15] Johnson T.R., Nguyen A., Shah K., Hogue G.D. (2019). Impact of insurance status on time to evaluation and treatment of meniscal tears in children, adolescents, and college-aged patients in the United States. Orthop J Sports Med.

[16] Deviandri R., Daulay M.C., Iskandar D., Kautsar A.P., Lubis A.M.T., Postma M.J. (2023). Health-economic evaluation of meniscus tear treatments: A systematic review. Knee Surg Sports Traumatol Arthrosc.

[17] Sochacki K.R., Varshneya K., Calcei J.G. (2020). Comparing meniscectomy and meniscal repair: A matched cohort analysis utilizing a national insurance database. Am J Sports Med.

[18] Feeley B.T., Liu S., Garner A.M., Zhang A.L., Pietzsch J.B. (2016). The cost-effectiveness of meniscal repair versus partial meniscectomy: A model-based projection for the United States. Knee.

[19] Schweizer C., Hanreich C., Tscholl P.M. (2021). Nineteen percent of meniscus repairs are being revised and failures frequently occur after the second postoperative year: A systematic review and meta-analysis with a minimum follow-up of 5 years. Knee Surg Sports Traumatol Arthrosc.

[20] Bae J.H., Kim J.G. (2021). Knee Arthroscopy: An Up-to-Date Guide.

[21] Spang R.C., Nasr M.C., Mohamadi A., DeAngelis J.P., Nazarian A., Ramappa A.J. (2018). Rehabilitation following meniscal repair: a systematic review. BMJ Open Sport Exerc Med.

[22] Abrams G.D., Frank R.M., Gupta A.K., Harris J.D., McCormick F.M., Cole B.J. (2013). Trends in meniscus repair and meniscectomy in the United States, 2005-2011. Am J Sports Med.

[23] Mahajan S., Caraballo C., Lu Y. (2021). Trends in differences in health status and health care access and affordability by race and ethnicity in the United States, 1999-2018. JAMA.

[24] Wehby G.L., Lyu W. (2017). The impact of the ACA Medicaid expansions on health insurance coverage through 2015 and coverage disparities by age, race/ethnicity, and gender. Health Serv Res.

[25] McMaughan D.J., Oloruntoba O., Smith M.L. (2020). Socioeconomic status and access to healthcare: Interrelated drivers for healthy aging. Front Public Health.

